# LIF–IGF Axis Contributes to the Proliferation of Neural Progenitor Cells in Developing Rat Cerebrum

**DOI:** 10.3390/ijms232113199

**Published:** 2022-10-30

**Authors:** Sho Takata, Hiromi Sakata-Haga, Hiroki Shimada, Tsuyoshi Tsukada, Daisuke Sakai, Hiroki Shoji, Mitsuhiro Tomosugi, Yuka Nakamura, Yasuhito Ishigaki, Hideaki Iizuka, Yasuhiko Hayashi, Toshihisa Hatta

**Affiliations:** 1 Department of Neurosurgery, Kanazawa Medical University, Kahoku 920-0293, Ishikawa, Japan; 2 Department of Anatomy, Kanazawa Medical University, Kahoku 920-0293, Ishikawa, Japan; 3 Department of Medical Science, Kanazawa Medical University, Kahoku 920-0293, Ishikawa, Japan; 4 Department of Neurosurgery, Saiseikai Toyama Hospital, Toyama 931-8533, Toyama, Japan; 5 Department of Biology, Kanazawa Medical University, Kahoku 920-0293, Ishikawa, Japan; 6 Department of Life Science, Kanazawa Medical University, Kahoku 920-0293, Ishikawa, Japan

**Keywords:** leukemia inhibitory factor, insulin-like growth factor 1, insulin-like growth factor 2, cerebrum, neural progenitor cell, neurogenesis, maternal immune activation, psychiatric disorders

## Abstract

In rodent models, leukemia inhibitory factor (LIF) is involved in cerebral development via the placenta, and maternal immune activation is linked to psychiatric disorders in the child. However, whether LIF acts directly on neural progenitor cells (NPCs) remains unclear. This study performed DNA microarray analysis and quantitative RT-PCR on the fetal cerebrum after maternal intraperitoneal or fetal intracerebral ventricular injection of LIF at day 14.5 (E14.5) and determined that the expression of insulin-like growth factors (IGF)-1 and -2 was induced by LIF. Physiological IGF-1 and IGF-2 levels in fetal cerebrospinal fluid (CSF) increased from E15.5 to E17.5, following the physiological surge of LIF levels in CSF at E15.5. Immunostaining showed that IGF-1 was expressed in the cerebrum at E15.5 to E19.5 and IGF-2 at E15.5 to E17.5 and that IGF-1 receptor and insulin receptor were co-expressed in NPCs. Further, LIF treatment enhanced cultured NPC proliferation, which was reduced by picropodophyllin, an IGF-1 receptor inhibitor, even under LIF supplementation. Our findings suggest that IGF expression and release from the NPCs of the fetal cerebrum in fetal CSF is induced by LIF, thus supporting the involvement of the LIF–IGF axis in cerebral cortical development in an autocrine/paracrine manner.

## 1. Introduction

Leukemia inhibitory factor (LIF), an IL-6 family cytokine, exhibits multifunctional activities, including induction of astrocyte differentiation and proliferation of neural progenitor cells (NPCs), maintenance of postmitotic neurons during development, and has therefore a key role in adult neurogenesis [1,2,3,4,5]. The LIF signal is transduced via gp130, a common receptor of the IL-6 cytokines family [6,7]. The downstream signaling of gp130 is mediated by the JAK-STAT3 pathway, which cooperates with Notch activation for neural stem cell self-renewal [8,9,10]. Our previous study showed that direct injection of recombinant LIF into the lateral ventricle of the cerebrum of mice at embryonic day 14.5 (E14.5) resulted in an increased number of neurons in the cerebral cortical plate (CP). However, the number of cells in CP significantly decreased in gp130-deficient mice [11]. In vitro, LIF increased the number of neurosphere-forming cells derived from the cerebrum of E14.5 mouse fetuses [4,12]. These results suggest that LIF plays a role in the proliferation of NPCs to form the fetal cerebral cortex.

We have previously reported that LIF levels in cerebrospinal fluid (CSF) exhibited a peak at E13 to E14 in mice [13] and at E15.5 in rats [14]. Interestingly, maternal LIF does not pass through the placenta but induces placental trophoblasts to produce the adrenocorticotropic hormone, which then promotes the secretion of LIF from fetal nucleated red blood cells, thus leading to a surge of LIF in the fetal CSF [14,15,16,17,18]. Finally, an increased level of LIF in fetal CSF enhanced NPC proliferation in the subventricular zone/ventricular zone (SVZ/VZ) at E15.5 in rats [14]. In contrast, maternal immune activation (MIA), caused by severe inflammation, caused a reduction in LIF in the CSF and impaired NPC proliferation in the SVZ/VZ in mouse fetuses [16,19]. Thus, LIF in the CSF participates in NPC proliferation in the developing cerebrum. However, the mechanism by which LIF promotes the proliferation of NPCs remains unclear. 

In the present study, we comprehensively searched for co-factors that are upregulated by LIF and cooperate with LIF to induce NPC proliferation in the fetal cerebrum and evaluated the participation of the obtained candidates in the LIF-dependent induction of NPCs proliferation. Our preliminary transcriptome analysis revealed that LIF induced insulin-like growth factor (IGF)-1 and IGF-2 (IGFs) in NPCs in the fetal cerebrum. In this context, we defined the relationship between LIF and IGFs and their involvement in neuronal secretion in the rat fetal cerebrum.

## 2. Results

### 2.1. LIF Upregulates the Expression of Igf1 and Igf2 in the Dorsal Cerebrum

**Figure 1:**
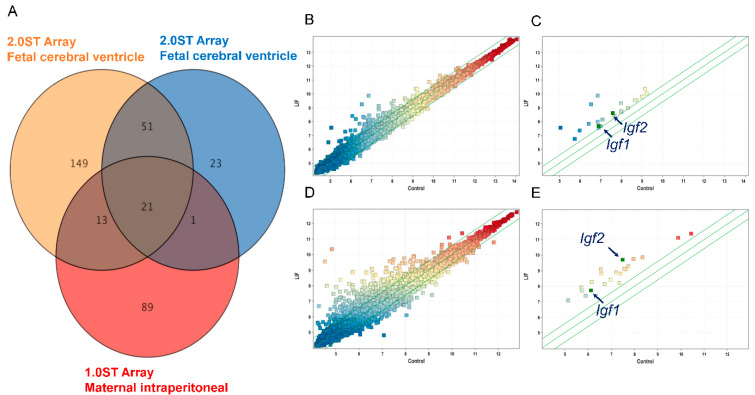
DNA microarray analysis in the fetal cerebrum at E14.5 after LIF injection into the peritoneal cavity of dams or cerebral ventricles of fetuses. LIF injection into dams intraperitoneally or fetal cerebrum ventricle directly increased *Igf1* and *Igf2* gene expressions in the fetal dorsal cerebrum at E14.5 rat fetuses: (**A**) The number of common differentially expressed genes (DEGs) between LIF injection into dams intraperitoneally and fetal cerebrum ventricle directly (|Fold Change (FC)| > 1.5) is shown. (**B**,**C**) the peritoneal injection to dams cohort included LIF- (n = 1) and saline- (n = 1, as control) injected animals. (**D**,**E**) The intracerebroventricular injection to fetuses cohort included LIF- (n = 2) and PBS- (n = 3, as control) injected animals. Among the upregulated DEGs, we focused on *Igf1* and *Igf2*. Pairwise scatter plots are shown, and 21 DEGs were identified based on the cut-off criteria (|Fold Change (FC)| > 1.5). The positions of *Igf1* and *Igf2* are indicated by black arrows.

**Figure 2:**
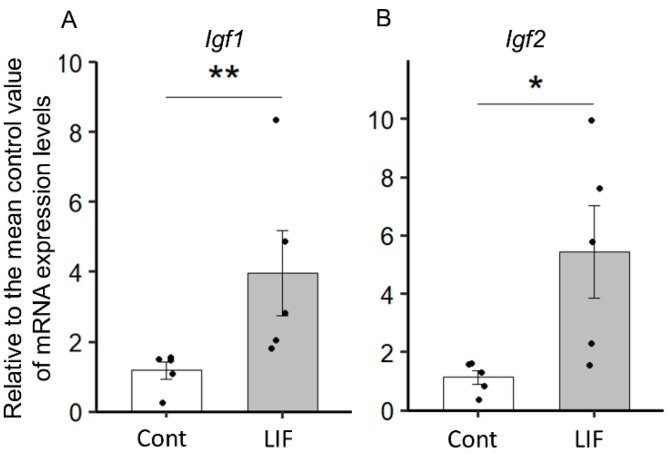
LIF injection into the fetal cerebral ventricle induces *Igf1* (**A**) and *Igf2* (**B**) mRNA expression in fetal dorsal cerebrum. Quantitative real-time PCR analyses revealed the increased expression of *Igf1* and *Igf2* in the fetal dorsal cerebrum upon direct administration of LIF into the lateral ventricle. Data are represented as ratios of the mean value of LIF-injected group (n = 5) versus control group (n = 5). Error bars: SEM. Statistical difference was determined by Mann–Whitney U test. * *p* < 0.05, ** *p* < 0.01.

We have previously demonstrated that LIF increased the proliferation of NPCs in the fetal cerebrum in both mice [13] and rats [14]. To ascertain the effect of LIF on the gene expression in the dorsal cerebrum, we performed a DNA microarray analysis of the dorsal cerebrum after LIF administration to either the maternal peritoneal cavity or directly to the lateral ventricle of the fetal cerebrum in utero. Based on the cut-off criteria (|Fold Change (FC)| > 1.5), 21 common differentially expressed genes (DEGs) were identified (Figure 1A, Table 1), including *Igf1* and *Igf2,* two neuronal growth factors. Pairwise scatter plots were also generated to visualize the gene level differences between LIF administration and control of each intraperitoneal injection group (Figure 1B,C) and intracerebroventricular injection group (Figure 1D,E). The effect of LIF on the expression of *Igf1* and *Igf2* in the dorsal cerebrum of rat fetuses was evaluated by quantitative RT-PCR analysis. *Igf1* and *Igf2* mRNA expression levels in the dorsal cerebrum were significantly increased in the LIF injected group compared with those in the control group at E14.5 (*Igf1*: *p* < 0.01; *Igf2*: *p* < 0.05, Mann–Whitney U test) (Figure 2). Overall, our DNA microarray analysis suggested that IGF-1 and IGF-2 contribute to LIF-dependent NPC proliferation. 

### 2.2. Physiological Change in IGF-1 and IGF-2 Levels in Fetal CSF

**Figure 3:**
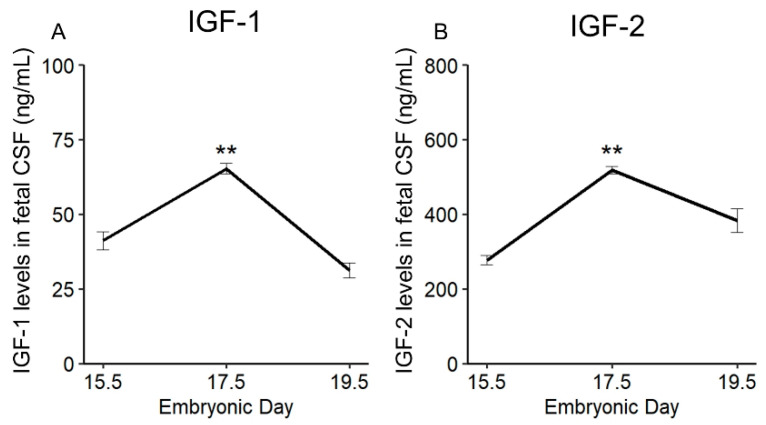
Physiological change in IGF-1 and IGF-2 levels in fetal cerebrospinal fluid (CSF). The levels of IGF-1 and IGF-2 in the CSF peaked at E17.5: (**A**) IGF-1: E17.5 vs. E15.5, *p* < 0.01; E19.5 vs. E17.5 *p* < 0.01. (**B**) IGF-2; E17.5-E15.5 *p* < 0.01; E19.5–E17.5 *p* < 0.01. Each group has three samples. Values are presented as mean ± SEM. One-way ANOVA followed by a Tukey post hoc test was used for statistical analysis. ** *p* < 0.01.

IGF-1 [20] and IGF-2 [21] have been well-known as the growth factors for NPCs in fetal CSF. First, we measured IGF-1 and IGF-2 levels in fetal CSF using an enzyme-linked immunosorbent assay (ELISA) at E15.5, E17.5, and E19.5. We found that following the surge of LIF levels in CSF, which was determined in our previous studies [13,14], levels of IGF-1 and IGF-2 in CSF peaked at E17.5 (IGF-1: *p* < 0.01, IGF-2: *p* < 0.01; IGF-1: E17.5 vs. E15.5 and E19.5, *p* < 0.01; IGF-2: E17.5 vs. E15.5 and E19.5, *p* < 0.01, one-way ANOVA followed by Tukey post hoc test) (Figure 3A,B). The present results are consistent with those described by Salehi et al. (2009) in mice [22], reporting that IGF-1 in fetal CSF of mice increased from day E16 to E18 and decreased from day E19 to E21.

### 2.3. Expression of IGF-1 and IGF-2 in the Cerebrum of Rat Fetuses

**Figure 4:**
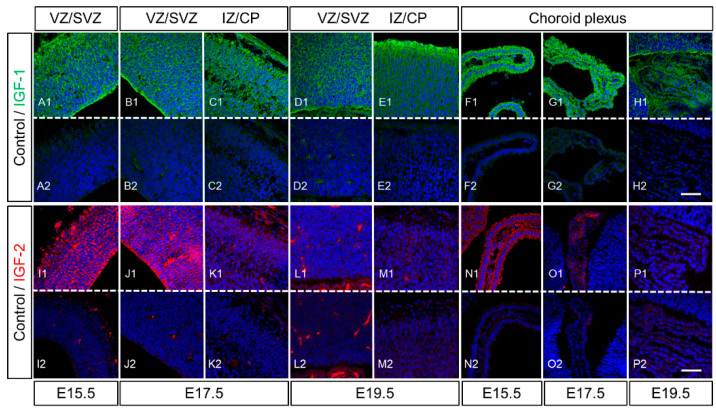
Expression of IGF-1 and IGF-2 in the fetal dorsal cerebrum during development. Expression of IGF-1 (green) was widely observed in the dorsal cerebrum and choroid plexus from E15.5 to E19.5 (**A1**–**H1**). On the other hand, IGF-2 (red) was observed in the dorsal cerebrum and choroid plexus at E15.5 and E17.5 (I1-K1) but drastically decreased at E19.5 (L1,M1). VZ/SVZ: ventricular zone/subventricular zone; IZ: intermediate zone; CP: cortical plate. The negative controls were stained with normal rabbit IgG for IGF-1 or mouse IgG for IGF-2 as primary antibodies (represented by (**A2**–**P2**) for panels (**A1**–**P1**), respectively). The nuclei were stained with Hoechst 33342 (blue). Scale bars: 50 µm.

Immunohistochemical analysis revealed that IGF-1 was widely distributed in the dorsal cerebrum, from the SVZ/VZ to the CP and epithelium of the choroid plexus, at E15.5 to E19.5 (Figure 4A–H). In particular, the NPCs of the apical and basal sides of the epithelium and choroid plexus showed strong intensities at E15.5 and E17.5. Unlike IGF-1, IGF-2 was expressed in the NPCs and choroid plexus epithelium of the cerebrum at E15.5 and E17.5 but rapidly decreased at E19.5, during the prepartum period (Figure 4I–P).

### 2.4. Colocalization of IGF-1 Receptor (IGF-1R) and Insulin Receptor α (IR-α) in the Cerebrum of Rat Fetuses

**Figure 5:**
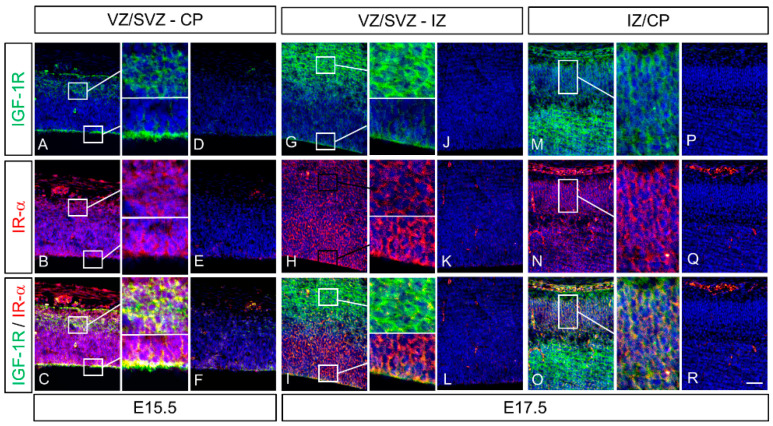
Expression of IGF-1 receptor (IGF-1R) and insulin receptor α (IR-α) in the fetal dorsal cerebrum at E15.5 and E17.5. IGF-1R (green) and IR-α (red) were widely expressed in the dorsal cerebrum at E15.5 and E17.5 (**A**,**B**,**G**,**H**,**M**,**N**). A strong intensified co-localization of IGF-1R and IR-α was observed, especially along the apical surface of the ventricular zone (VZ) and intermediate zone (IZ) (**C**,**I**,**O**). The negative controls were stained with normal rabbit IgG (**D**–**F**,**J**–**L**,**P**–**R**), respectively). Colocalization of these receptors is denoted in yellow. The nuclei were stained with Hoechst 33342 (blue). Scale bar: 50 µm.

To investigate the role of IGF-1 and IGF-2, induced by LIF, in the development of the fetal rat cerebral cortex, we analyzed the spatial and temporal expression patterns and co-localization of IGF-1R and IR-α, the receptors to which IGF-1 and IGF-2 bind, in the fetal mouse cerebrum using fluorescence double immunostaining. IGF-1 and IR-α were expressed in VZ/SVZ, IZ, and CP at E15.5 and E17.5 (Figure 5). Colocalized strong intensities of IGF-1R and IR-α were determined, especially in the VZ and IZ (Figure 5C,I). 

### 2.5. Colocalization of IGF-1R, IR-α, LIF Receptor (LIFR), and gp130 in the Cerebrum and Choroid Plexus of Rat Fetuses

**Figure 6:**
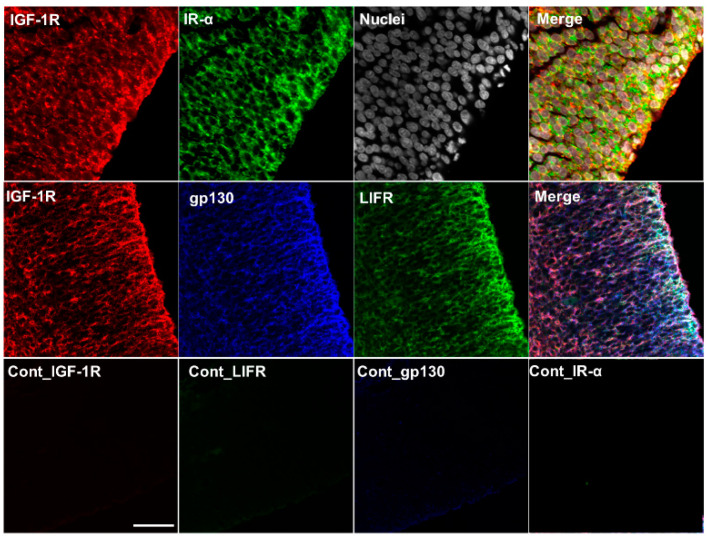
Localization of IGF-1R, IR-α, LIFR, and gp130 in the fetal dorsal cerebrum at E15.5. The localization of IGF-1R is denoted in red, IR-α in green, and their synthesis in yellow (upper raw). Strong staining intensity of IGF-1R (red), gp130 (blue), and LIFR (green) was observed along the apical (ventricular) side of the ventricular zone. The co-localization of these three receptors is demonstrated in white (merge, middle raw). The nuclei were stained with Hoechst 33342 (gray). Cont: negative controls stained with non-immunized rabbit IgG. Scale bar: 25 µm.

**Figure 7:**
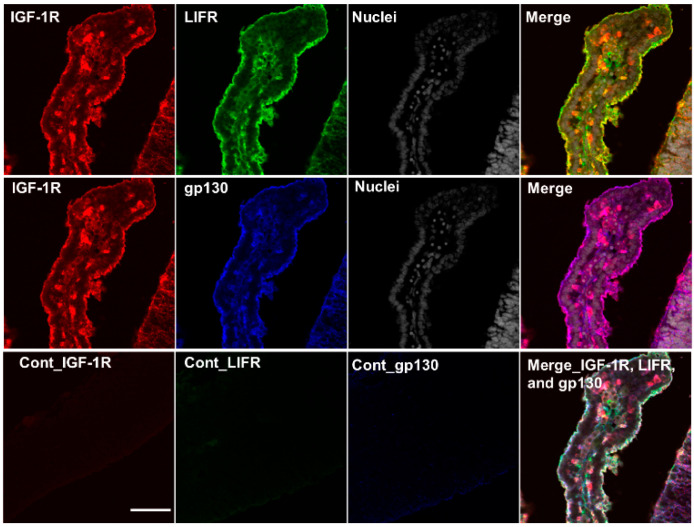
Localization of IGF-1R, LIFR, and gp130 in the choroid plexus of the lateral ventricle at E15.5. Localization of IGF1-R (red), LIFR (green), and gp130 (blue) was observed in the apical side of the choroid plexus epithelium. The co-localization of all three receptors is shown in white. The nuclei were stained with Hoechst 33,342 (gray). Cont: negative controls stained with non-immunized rabbit IgG, respectively. Scale bar: 25 µm.

The induction of IGF-1 and IGF-2 expression in the NPCs of the dorsal cerebrum by LIF stimulation was confirmed through experiments comprising maternal LIF administration (Figure 1 and Table 1) and direct LIF administration to the fetal ventricles (Figure 2). The co-expression of LIFR, gp130, IGF-1R, and IR-α, and the receptors for IGFs is pertinent to the contribution of the LIF–IGF circuit to neurogenesis in NPCs, which was examined using multiplex fluorescent immunostaining. IGF-1R and IR-α were highly expressed in the dorsal cerebrum, and their co-localization was determined (Figure 5, upper raw). Colocalization of IGF-1R, gp130, and LIF was also determined, showing an intense signal along the ventricular surface (Figure 6, middle raw). As shown in Figure 4, the choroid plexus is another potential source of IGF-1 and IGF-2 in the CSF. To investigate the possible induction of IGFs by LIF from the choroid plexus in the lateral ventricles, expression of IGF-1R, LIFR, and gp130 was also examined in the choroid plexus in the lateral ventricle at E15.5, and the co-expression of these receptors was determined in the epithelia (Figure 7). 

### 2.6. LIF-Induced Proliferation of NPCs Is Dependent on IGF-1 or IGF-2

**Figure 8:**
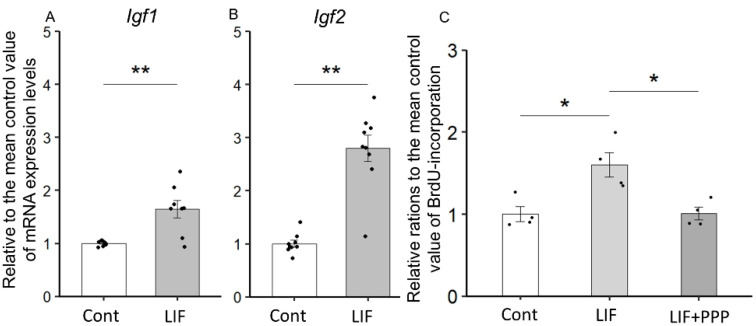
Induction of *Igf1* and *Igf2* mRNA by LIF and inhibition of LIF-induced proliferation in neural progenitor cells (NPCs) with IGF-1R inhibitor: (**A**,**B**) quantitative RT-PCR analysis showed that LIF supplementation in culture media increased *Igf1* and *Igf2* mRNA in NPCs. *Igf1:* n = 8 per group, *Igf2:* n = 9 per group. (**C**) LIF-induced proliferation was assessed with a 5-Bromo-2′-deoxyuridine (BrdU)-incorporation assay and revealed that the increase in LIF-dependent BrdU incorporation was inhibited by the addition of picropodophyllin (PPP), an inhibitor of IGF-1R. Data are presented as relative ratios to the mean value of the control (Cont). Each group has four samples. LIF vs. Cont, *p* < 0.05; LIF vs. LIF + PPP *p* < 0.05. Statistical analysis was performed with the Kruskal–Wallis test followed by Mann–the Whitney U test. Error bars: SEM. * *p* < 0.05, ** *p* < 0.01.

The co-localization of IGF-1R, IR-α, gp130, and LIFR in the NPCs of VZ at E15.5 (Figure 6) suggests that LIF-induced IGF-1 and IGF-2 stimulate the proliferation of NPCs in an autocrine/paracrine manner. Using neurospheres generated from the dorsal cerebrum of E14.5 rat fetuses, we examined whether IGF-1 and IGF-2 production induced in NPCs by LIF treatment is involved in the LIF-dependent mitogenic effects on NPCs. First, the expression levels of *Igf1* and *Igf2* increased in the LIF-treated neurospheres (*p* < 0.01, Mann–Whitney U test) (Figure 8A,B). We next examined whether LIF promoted NPCs proliferation via induction of IGF-1 and IGF-2 using a 5-Bromo-2′-deoxyuridine (BrdU)-incorporation assay. Increased BrdU incorporation in NPCs was observed in the LIF-treated group compared with that in the control group with primary medium (*p* < 0.01, Mann–Whitney U test) (Figure 8C). Furthermore, to confirm that the proliferative effect of LIF on NPCs is mediated by IGF-1 and/or IGF-2, picropodophyllin (PPP), an inhibitor of tyrosine phosphorylation of IGF-1R, was added to the culture medium [23]. A reduction in BrdU incorporation in NPCs under LIF-supplemented conditions was observed upon the addition of PPP (*p* < 0.01, Kruskal–Wallis test followed by Mann–Whitney U test) (Figure 8C).

## 3. Discussion

In a previous study, we showed that fetal-maternal LIF signal relay upregulates LIF levels in fetal serum and CSF and induces NPCs proliferation in the developing cerebrum of rodents [14]. However, whether LIF induces proliferation in NPCs directly or indirectly through other effectors remains unclear. In the present study, differential expression analysis of the fetal cerebrum after LIF administration identified 21 upregulated genes, among which *Igf1* and *Igf2*, which are neuronal growth factors [21,22]. Interestingly, following the physiological surge of LIF in the CSF at E15.5 of rat fetuses [14], the physiological levels of IGF-1 and IGF-2 in the CSF peaked at E17.5 (Figure 3A). Furthermore, an increase in *Igf1* and *Igf2* mRNA expression in the fetal dorsal cerebrum was observed upon direct LIF injection into the cerebral ventricles of fetuses. Moreover, LIF induced *Igf1* and *Igf2* expression in neurospheres generated from the dorsal cerebrum of E14.5 rat fetuses, suggesting that NPCs are the source of IGF-1 and IGF-2 in the CSF. In many cell types, IGF-1 and IGF-2 have diverse biological activities, including induction of cell proliferation, differentiation, and cell survival [24]. Most NPCs and postmitotic neurons in the fetal forebrain express IGF-1 and IGF-1R, contributing to the proliferation of NPCs and survival of neurons in an autocrine/paracrine manner [25,26,27,28]. In rodents, IGF-2 is highly expressed in fetuses, namely the placenta and brain [29]. Consequently, it dramatically decreases after birth. Eventually, in adult rodents, it is expressed only in the choroid plexus and meninges [30]. Conversely, IGF-2 is highly expressed in various organs in adult humans [31]. The drastic alteration of the IGF-2 expression pattern in the perinatal period in rodents is highly distinctive from that in humans. These reports coincide with the results of this study. 

Mutations of *Igf1* in humans lead to severe growth and mental retardation [32]. Furthermore, *Igf1*-deficient mice exhibited low birth weight and had severe developmental retardation after birth [33,34]. The *Igf2*-deficient mice also showed growth retardation at birth, similar to that of *Igf1*-deficient mice [30,34], indicating that both IGF-1 and IGF-2 contribute to fetal development. IGF-1R has a high affinity for IGF-1 and IGF-2. IR-α, an insulin receptor RNA splicing variant, has a high affinity for both insulin and IGF-2 and is expressed in fetuses [35]; IGFs bind to a hybrid form of IR-α along with IGF-1R with a higher affinity than that of insulin [24,35,36]. Immunohistochemical analysis showed the localization of IGF-1 and IGF-2 in the dorsal cerebrum and choroid plexus over E15.5, 17.5, and 19.5 (Figure 4, Figure 5, Figure 6 and Figure 7); IGF-1 and IGF-2 levels in CSF peaked at E17.5 (Figure 3). Our previous study revealed that LIF levels in the fetal rat CSF peaked at around E15 [14], preceding that of the IGFs revealed in this study. These results indicate that the LIF–IGF axis contributes to fetal brain development as part of the LIF signaling relay. Furthermore, immunostaining revealed the co-localization of IGF-1R, IR-α, LIFR, and gp130 on the apical side of the VZ, further supporting the existence of a LIF–IGF axis from the CSF to NPCs (Figure 5 and Figure 6).

An induction of *Igf1* and *Igf2* mRNA expression by LIF both in the fetal dorsal cerebrum and cultured NPCs derived from the dorsal cerebrum indicates that the parenchyma of the cerebrum was one of the main sources of IGF-1 and IGF-2 in the fetal CSF. In the neurosphere assay, LIF treatment increased the expression of *Igf1* and *Igf2* mRNA in NPCs (Figure 8A,B), and NPCs proliferation was also increased, both of which were inhibited by PPP, an IGF-1R inhibitor (Figure 8C). These findings suggest that IGF-1R mediates the mitogenic effect of LIF on NPCs. PPP showed a specific inhibitory effect on IGF-1R by blocking tyrosine phosphorylation without affecting IR or other receptors contributing to proliferation/differentiation, such as fibroblast growth factor receptor or epidermal growth factor receptor [37]. In this study, co-localization of IR-α and IGF-1R was determined in NPCs by immunostaining (Figure 5 and Figure 6). The present immunostaining findings of IGF-1R and IR-α in the NPCs suggest that both IGF-1 and IGF-2 contribute to neuron production in the NPCs of the fetal rat brain via IGF-1R and IR-α. Further analysis using either siRNA or neutralizing antibodies against IGF-1, IGF-2, or IR-α in combination with PPP is needed to better decipher the exact role of LIF-induced IGFs in NPCs proliferation.

The present study was based on the described involvement of maternal LIF in the unique neuro-immune-endocrine network between mother and fetus and its role in fetal cortical development, as reported by Simamura et al. (2010) [14]. It can be presumed that the fetal-maternal LIF signal relay induced the secretion of IGF-1 and IGF-2 in the fetal cerebrum in an autocrine/paracrine manner, thus contributing to cerebral cortical development. IGF signaling regulates the PI3K/Akt pathway and the differentiation and maturation of neurons generated from NPCs [20,38]. Of note, IGF-1 also has an essential role in the migration of GABAergic and glutamatergic neurons in early development related to higher brain function [39]. The basis for the development of autism spectrum disorder (ASD), one of the neurodevelopmental disorders, has been shown to involve improper developmental processes such as neurogenesis, migration, neurite growth, synaptogenesis, synaptic plasticity [40], and MIA, which represents a potential risk factor [41]. LIF belongs to the IL-6 cytokines family, which has a common receptor gp130 [6,7]. Therefore, under inflammatory conditions, LIF and IL-6 competitively share gp130. Excess IL-6 signaling via JAK/STAT3 in placental trophoblasts causes ASD in mice offspring [42]. Recently, it has been reported that the size of some cortices, including the somatosensory cortex, decreased in offspring exposed to MIA [43]. Our previous study also showed that MIA caused a substantial surge in IL-6 levels in the maternal serum and a reduction in LIF levels in the fetal CSF, resulting in a decrease in cell number and volume in the fetal cerebrum [16]. A disturbed CSF-borne LIF–IGF axis might play a role in the mechanism of MIA-induced cortex reduction. Thus, impaired expression of IGFs in fetuses under adverse conditions in utero leads to a possible risk of neurological disorders of prenatal origin, including ASD. The present findings may provide significant advances in the research on the developmental origins of health and disease (DOHaD) [44].

## 4. Materials and Methods

### 4.1. Animals

Female Wistar Hannover rats aged 12–24 weeks were used in this study (SLC Japan, Inc., Hamamatsu, Japan). The rats were maintained under standard laboratory conditions with ad libitum food and water intake. A female rat was housed with a male overnight, and the day on which a vaginal plug was found in the morning was marked as E0.5. All procedures in this study were performed according to the Care and Use of Laboratory Animals of Kanazawa Medical University, Kanazawa, Japan. The protocol was approved by the Kanazawa Medical University Institutional Animal Care and Use Committee (protocol numbers 2017-9, 2020-52). The procedure was performed using an anesthetic combination of midazolam (4 mg/kg BW), medetomidine (0.3 mg/kg BW), and butorphanol (5 mg/kg BW), and all efforts were made to minimize animal suffering.

### 4.2. Induction of IGF-1 and IGF-2 in the Fetal Cerebrum by LIF

According to our previous study, the serum level of LIF reached its peak at E14.5 in pregnant dams of rats, and maternally injected LIF induced NPCs proliferation in the fetal cerebrum in rats [14]. Therefore, we injected 5 μg/kg body weight (BW) recombinant rat LIF (3010, MilliporeSigma, Burlington, MA, USA) into pregnant dams intraperitoneally at E14.5 to analyze total mRNA expression using DNA microarray and *Igf1* and *Igf2* expression using quantitative RT-PCR. In addition, we also examined the effects of LIF directly injected into the lateral ventricle of the fetal cerebrum at E14.5 via in utero injection technique [38]. For in utero injection, 1 μL of recombinant rat LIF (5 ng/μL) diluted with PBS and Fast Green (100:1, FUJIFILM Wako Pure Chemical, Osaka, Japan) was administered to each fetus. The same volume of PBS was injected into the lateral ventricle of control fetuses. Three hours after the injection, the dorsal cerebrum was collected and stored as a pooled sample for each group.

### 4.3. Sample Preparation of Fetal CSF and Serum

Under the same anesthetic conditions for dams, fetuses at E15.5, E17.5, and E19.5 were dissected; a pooled sample of fetal blood was collected from the heart, and sera were isolated [13,14]. Then, a pooled sample of fetal CSF via the fourth ventricle was collected. Three dams on each embryonic day were used for the sample collection. The collected fetal CSF and serum were stored at −30 °C with Protein Stabilizing Cocktail^®^ (Thermo Fisher Scientific, Waltham, MA, USA) for further studies.

### 4.4. RNA Preparation

The dorsal cerebrums of the fetuses were cut into pieces and instantly stored in RNAlater^®^ (Thermo Fisher Scientific, Waltham, MA, USA). After incubation for 24 h, the RNAlater^®^ was discarded, and the specimens were stored at −80 °C until use. Total RNA was extracted from brain tissue using ISOGEN II (Nippon Gene Co., Ltd., Tokyo, Japan). Total RNA from cultured cells was extracted using the RNeasy Micro Kit (Qiagen, Germantown, MD, USA). Genomic DNA was digested using the DNase-I enzyme, according to the manufacturer’s instructions (Nippon Gene Co., LTD., Tokyo, Japan). The quality and concentration of extracted RNA were assessed using a DS-11 NanoPad (DeNovix, Wilmington, DE, USA). For the DNA microarray analysis, RNA quality was evaluated using a 2100 Bioanalyzer (Agilent Technologies, Inc., Santa Clara, CA, USA), generating an RNA integrity number (RIN). 

### 4.5. DNA Microarray and Real-Time Quantitative PCR 

Gene expression of the fetal cerebrum after injection of either LIF (n = 1) or saline (n = 1) in dams at E14.5 was analyzed using the Affymetrix GeneChip Rat Gene 1.0 ST Array (901172, Thermo Fisher Scientific, Waltham, MA, USA). Gene expression in the fetal cerebrum upon direct injection of LIF into the lateral ventricle of fetuses was also analyzed at E14.5 (LIF: n = 2, PBS: n = 3) using the Affymetrix GeneChip Rat Gene 2.0 ST Array (902124, Thermo Fisher Scientific, Waltham, MA, USA). For DNA microarray analysis, DEGs between LIF and control (saline or PBS) groups were identified, and candidate genes were selected based on a |Fold Change (FC)| > 1.5. The data were analyzed using Genespring 14.9.1 (Agilent, Santa Clara, CA, USA).

For quantitative RT-PCR, first-strand complementary DNA templates were synthesized with SuperScript VILO Reverse Transcriptase (Thermo Fisher Scientific, Waltham, MA, USA). Quantitative PCR was conducted using GeneAce SYBR^®^ qPCR Mix α (Nippon Gene Co., Ltd., Tokyo, Japan), and 18S ribosomal RNA was used as the internal control. The following primer sequences were used in this study: *18S ribosomal RNA* forward 5′-ACGGCTACCACATCCAAGGA-3′ and *18S ribosomal RNA* reverse: 5′-CGGGAGTGGGGTAATTTGCGPCR-3′, *Igf1* forward: 5′-GCTTTTACTTCAACAAGCCCACA-3′and *Igf1* reverse: 5′-TCAGCGGAGCACAGTACATC-3′; *Igf2* forward: 5′-AGATAGCCATGGGCAGCGTCGCCGGCTTCCAG-3′ and *Igf2* reverse: 5′-AGATAGCCCGGGTCACTGATGGTTGCTGGACATCTC-3′. PCR was conducted in duplicate using the Thermal Cycler Dice^®^ Real-Time System (TaKaRa Bio Inc., Kusatsu, Japan) and quantified using the delta–delta-cycle threshold method (2^−ΔΔCt^). Dissociation curve analysis was performed to confirm the specificity of the PCR products.

### 4.6. ELISA

To determine the physiological level of IGF-1 in fetal CSF and serum, the Mouse/Rat IGF-I/IGF-1 Quantikine ELISA Kit (MG100, R&D Systems, Minneapolis, MN, USA) was used. Following the manufacturer’s protocol, we estimated the IGF-1 levels in pooled fetal CSF or serum by measuring the absorbance at a wavelength of 450 nm using a 2104 EnVision (PerkinElmer, Waltham, MA, USA). The measurement of the pooled samples on each embryonic day was repeated three times. Fab fragments prepared from the anti-IGFII monoclonal antibody (mAb) (05-166, MilliporeSigma, Burlington, MA, USA) using Pierce™ Fab Micro Preparation Kit (Thermo Fisher Scientific) were labeled with biotin using EZ-Link NHS-biotin (20217, Thermo Fisher Scientific, Waltham, MA, USA) at a constant concentration of 97 μg/mL according to the manufacturer’s protocol. The pooled sample of either CSF or serum was diluted in PBS (1:300), and 100 μL of the diluted sample was applied to each well of an Immobilizer Amino plate (436007, Thermo Fisher Scientific, Waltham, MA, USA) to capture the amino residues of proteins/peptides and incubated at 4 °C overnight. After a four-time brief wash with TTBS, the wells were blocked with 100 μL of StartingBlock blocking buffer Thermo Fisher Scientific, Waltham, MA, USA ) at room temperature (RT) for 20 min, then incubated with biotin-labeled anti-IGF2 mAb (180 ng/mL) in TTBS at 4 °C, overnight. Finally, biotin-labeled anti-IGF2 Fab was detected with HRP-ExtrAvidin peroxidase (Sigma-Aldrich, Saint Louis, MO, USA) and SuperSignal ELISA pico (Thermo Fisher Scientific, Waltham, MA, USA), and chemiluminescence signals were measured using 2104 EnVision (PerkinElmer, Waltham, MA, USA). 

### 4.7. Fluorescence Immunohistochemistry

At E15.5, 17.5, and 19.5, fetuses were fixed with 4% paraformaldehyde in 50 mM PB (163-20145, FUJIFILM Wako Pure Chemical, Osaka, Japan) by immersion at 4 ℃ overnight, then specimens were embedded in optimal cutting temperature compound and stored at −80 ℃ until use. Serial coronal sections were cut at 20 μm and adhered to glass slides. After a brief wash with PBS containing 0.02% Triton X-100 (PBST), sections were microwaved in 10 mM citrate buffer (pH 6.0) for antigen retrieval, then incubated in a blocking solution containing 10% normal goat serum (Jackson ImmunoResearch, West Grove, PA, USA) diluted in PBST for 1 h at RT 1%. Sections were incubated with either rabbit anti-IGF-1 polyclonal antibody (pAb) (1 µg/mL; ab9572, abcam, Cambridge, UK), mouse anti-IGF-2 mAb (10 µg/mL; 05-166, MilliporeSigma, Burlington, MA, USA), rabbit anti-IGF-1 receptor pAb (2 µg/mL; ab131476, abcam, Cambridge, UK), or mouse anti-insulin receptor α mAb (2 µg/mL; sc-57344, Santa Cruz, Dallas, TX, USA) in blocking solution at 4 °C overnight. Non-immunized rabbit IgG (1 µg/mL; X0903, DAKO, Santa Clara, CA, USA) for IGF-1 or mouse IgG (10 µg/mL; X0931, DAKO, Santa Clara, CA, USA) for IGF-2 was used as a negative control. 

Sections were washed with PBST and detected using Alexa Fluor 594 goat anti-rabbit IgG (1 µg/mL; Thermo Fisher Scientific, Waltham, MA, USA) or Alexa Fluor 594 goat anti-mouse IgG H&L (1 µg/mL; Thermo Fisher Scientific, Waltham, MA, USA). To determine the co-localization of the IGF-1R, IR-α, LIFR, and gp130 in the cerebrum at E15.5, multiplex fluorescent immunostaining using the Opal™ 4-Color Manual IHC Kit (PerkinElmer, Waltham, MA, USA) was performed following the manufacturer’s instructions. Serial coronal sections of E15.5 fetuses cut at 15 μm were used. The primary antibodies used included rabbit anti-IGF-1 receptor pAb (2 µg/mL; ab131476, abcam, Cambridge, UK), mouse anti-insulin receptor α mAb (2 µg/mL; sc-57344, Santa Cruz), rabbit anti-LIFR pAb (1 µg/mL; sc-515337, Santa Cruz, Dallas, TX, USA,), and rabbit anti-gp130 pAb (1 µg/mL; sc-22346, Santa Cruz, Dallas, TX, USA ). Envision + System-HRP Labelled Polymer Anti-Rabbit or Mouse (Agilent Technologies, Santa Clara, CA, USA) was used as a secondary antibody. Non-immune rabbit IgG (Sigma-Aldrich, Saint Louis, MO, USA) was used as a negative control. Hoechst 33342 (5 µg/mL; H3570, Thermo Fisher Scientific, Waltham, MA, USA) containing RNase A (10 µg/mL; 313-02821, NIPPON GENE, Tokyo, Japan,) was used for nuclear staining. Images were captured using an LSM 710 confocal microscope (ZEISS, Oberkochen, Germany).

### 4.8. Neurosphere Assay 

Neurospheres were derived from mechanically dissociated cells obtained from the dorsal cerebrum at E14.5. Neurospheres were cultured in basal serum-free media Neurobasal Medium (21103049, Thermo Fisher Scientific, Waltham, MA, USA) containing 2% B27 supplement (17504044, Thermo Fisher Scientific, Waltham, MA, USA), 1% glutamine (35050061, Thermo Fisher Scientific, Waltham, MA, USA), 1% antibiotic-antimycotic (15240096, Thermo Fisher Scientific, Waltham, MA, USA), 20 ng/mL recombinant human basic fibroblast growth factor (bFGF, 100-18B; Peprotech, Cranbury, NJ, USA), and 20 ng/mL epidermal growth factor (EGF, 315-09; PeproTech, Cranbury, NJ, USA) in culture flasks at a density of 2 × 10^5^ cells/mL at 37 ℃ in humidified air supplemented with 5% CO_2_ until use. The medium was replenished every 72 h. After six days of culture, floating neurospheres were dissociated using StemPro Accutase (A1110501, Thermo Fisher Scientific, Waltham, MA, USA). For proliferation studies, 2 × 10^5^ cells/mL were plated in 96-well (100 µL/well) round plates and cultured for 48 h with 5% CO_2_ and 5% O_2_. Then, 100 ng/mL LIF in each well was added to the treatment group.

To examine mRNA expression, eight wells were assessed per condition after 3 h. To evaluate the effects of LIF in association with IGFs on the proliferation of NPCs, a 5- Bromo-2′deoxyuridine (BrdU) ELISA kit (Roche, Indianapolis, IN, USA) was used, according to the manufacturer’s protocol. Briefly, 10 µM BrdU labeling reagent was added to each well, and the cells were incubated for 2 h. BrdU incorporation was quantified by a colorimetric immunoassay at 450 nm using 2104 EnVision (PerkinElmer, Waltham, MA, USA). To assess the contribution of IGF-1, which was secreted from LIF-stimulated neurospheres, to the proliferation of NPCs, 50 nM picropodophyllin (PPP, 407247-1MGCN, MilliporeSigma, Burlington, MA, USA), an IGF-1R inhibitor, was added to each well and BrdU incorporation was measured. A basic medium was used as a control.

### 4.9. Statistical Analyses

Data are presented as mean ± standard error of the mean (SEM). All statistical analyses were performed using R 4.0.0 (https://www.r-project.org, accessed on 21 August 2021). Statistical analysis was performed using the nonparametric Mann–Whitney U test if variances were not equal and parametric; for example, one-way ANOVA followed by Tukey’s post hoc test was employed if variances were equal. Levels of significance: * *p*  <  0.05, ** *p*  <  0.01, for all statistics in this study.

## 5. Conclusions

This study revealed that LIF induced IGF-1 and IGF-2 expression in NPCs and secretion into the CSF, which promoted the proliferation of neural progenitors via IGF-1R and IR-α in an autocrine/paracrine manner in rat fetuses.

## Figures and Tables

**Table 1:** Extracted differentially expressed 21 genes with fold change (FC) > 1.5 from the dataset of DNA microarray analysis screened by Pairwise scatter plots.

Gene Symbol	Gene Expression
	(LIF/Control)
*Igf2*	3.901060583
*Dcn*	3.608841573
*Egfl6*	3.442054291
*Pdgfra*	3.115182075
*Gpc3*	2.970747151
*Dab2*	2.795991831
*Bgn*	2.752705096
*Postn*	2.728830926
*Ddr2*	2.680990235
*Serpinf1*	2.672903839
*Lum*	2.62812436
*Anxa6*	2.566506249
*Ogn*	2.512830911
*Ror1*	2.450680042
*Anxa2*	2.421598147
*Igf1*	2.401063962
*H19|Mir675*	2.28898765
*Ctsc*	2.224476544
*Lgals1*	2.174736479
*S100a11*	1.98069301
*Colec12*	1.892572836

## Data Availability

The datasets generated and/or analyzed during the current study are not publicly available but may be available from the corresponding author upon reasonable request.
